# Patterns in the Use of Heart Failure Telemonitoring: Post Hoc Analysis of the e-Vita Heart Failure Trial

**DOI:** 10.2196/41248

**Published:** 2023-01-31

**Authors:** Maaike Brons, Iris ten Klooster, Lisette van Gemert-Pijnen, Tiny Jaarsma, Folkert W Asselbergs, Marish I F J Oerlemans, Stefan Koudstaal, Frans H Rutten

**Affiliations:** 1 Department of Cardiology University Medical Center Utrecht Utrecht Netherlands; 2 Department of Psychology, Health and Technology Center for eHealth Research and Disease Management University of Twente Enschede Netherlands; 3 Department of Nursing Science Julius Center for Health Sciences and Primary Care University Medical Center Utrecht, Utrecht University Utrecht Netherlands; 4 Department of Health, Medicine and Care Linköping University Linköping Sweden; 5 Department of Cardiology University of Amsterdam Amsterdam University Medical Centers Amsterdam Netherlands; 6 Health Data Research UK and Institute of Health Informatics University College London London United Kingdom; 7 Department of General Practice Julius Center for Health Sciences and Primary Care University Medical Center Utrecht, Utrecht University Utrecht Netherlands

**Keywords:** heart failure, telemonitoring, adherence, eHealth, remote monitoring, electronic personal health record, patient monitoring

## Abstract

**Background:**

Research on the use of home telemonitoring data and adherence to it can provide new insights into telemonitoring for the daily management of patients with heart failure (HF).

**Objective:**

We described the use of a telemonitoring platform—including remote patient monitoring of blood pressure, pulse, and weight—and the use of the electronic personal health record. Patient characteristics were assessed in both adherent and nonadherent patients to weight transmissions.

**Methods:**

We used the data of the e-Vita HF study, a 3-arm parallel randomized trial performed in stable patients with HF managed in outpatient clinics in the Netherlands. In this study, data were analyzed from the participants in the intervention arm (ie, e-Vita HF platform). Adherence to weight transmissions was defined as transmitting weight ≥3 times per week for at least 42 weeks during a year.

**Results:**

Data from 150 patients (mean age 67, SD 11 years; n=37, 25% female; n=123, 82% self-assessed New York Heart Association class I-II) were analyzed. One-year adherence to weight transmissions was 74% (n=111). Patients adherent to weight transmissions were less often hospitalized for HF in the 6 months before enrollment in the study compared to those who were nonadherent (n=9, 8% vs n=9, 23%; *P*=.02). The percentage of patients visiting the personal health record dropped steadily over time (n=140, 93% vs n=59, 39% at one year). With univariable analyses, there was no significant correlation between patient characteristics and adherence to weight transmissions.

**Conclusions:**

Adherence to remote patient monitoring was high among stable patients with HF and best for weighing; however, adherence decreased over time. Clinical and demographic variables seem not related to adherence to transmitting weight.

**Trial Registration:**

ClinicalTrials.gov NCT01755988; https://clinicaltrials.gov/ct2/show/NCT01755988

## Introduction

The COVID-19 pandemic has put pressure on health care systems worldwide, and it sparked new interest in home telemonitoring. In heart failure (HF) care, it could help monitor HF signs and symptoms, reduce face-to-face consultations, and improve patient empowerment [1]. It may include remote patient monitoring or an electronic personal health record or a combination of remote patient monitoring and an electronic personal health record [2]. A personal health record is an electronic application through which individuals can access, manage, and share their health information in a private, secure, and confidential environment [3]. A personal health record may also include self‐management support, patient‐provider communication, information about illness, peer support, or monitoring health behavior data [4].

Randomized trials evaluating the effectiveness of noninvasive telemonitoring in HF, with HF hospitalization or death as end point, were either neutral or positive. Different results can be explained, at least partly, by the variety in telemonitoring approaches used and the level of usual care in the comparator arm [5-7]. Furthermore, blood pressure and heart rate are often captured in conjunction with weight in telemonitoring systems, but the additional prognostic potential of daily measurements of these biometric values in providing information on upcoming hospitalizations for worsening HF has not been explored thoroughly [8]. On the other hand, several research groups reported that simple rules of sudden weight change in patients with HF demonstrated to generate many alerts with poor sensitivity, and therefore, remote patient monitoring of weighing alone seems of limited value [9-12]. However, these results may be driven by insufficient or inadequate use of telemonitoring by patients. These studies lacked reporting on the daily use and adherence of participants to telemonitoring. In addition, both applications (ie, remote patient monitoring and personal health record) are rarely used in trials, while this can provide valuable new insights in understanding how patients want to use telemonitoring systems [13].

We already know that a small number of patients will not use telemonitoring at all (2%-14%) and that, in general, the use of telemonitoring decreases over time [14-16]. Studies that reported on adherence to telemonitoring in HF mainly focused on adherence to the number of biometric measurements per week [14,15]. Less is known about the relation between patient and their characteristics, the level of adherence to remote patient monitoring, and the use of a personal health record in a single telemonitoring system. The analysis of log data (ie, actual and continuous information about real-time usage behavior of a noninvasive telemonitoring device) can provide objective insights into the actual use and adherence to remote patient monitoring [17]. Deterioration of HF may lead to rapid weight gain as a consequence of fluid retention, and if uncorrected, it can lead to hospitalization and ultimately death. Obviously, weight management is important, and it is recommended by the European Society of Cardiology that patients should be trained to self-adjust their diuretic dose based on monitoring of signs or symptoms of HF deterioration and daily weight measurements [18].

The aim of this study was to quantify the use of telemonitoring (both remote patient monitoring and personal health record) in patients with stable HF and to assess whether patient characteristics were related to adherence or nonadherence to weight transmissions.

## Methods

### Procedure

We used the data of the e-Vita HF study. The design and results of the e-Vita HF study were reported elsewhere [1,19]. In short, the e-Vita HF study was a 3-arm parallel randomized trial in patients with stable chronic HF (337/450, 75% of the entire study population was self-assessed New York Heart Association [NYHA] class I or II) who were managed for at least 3 months in one of 9 heart failure outpatient clinics in the Netherlands; the study compared an eHealth-adjusted care pathway with (1) usual care and (2) usual care plus guided access to the heartfailurematters.org website [1,19]. Patients were followed up for 1 year. Patients were individually randomized by computerized block randomization (maximum of 9 patients per block) to one of the 3 groups (150 patients in each group) [1,19].

The eHealth-adjusted care pathway included an interactive platform for HF disease management with (1) a remote patient monitoring facility for weight, blood pressure, and pulse; and (2) a personal health record [1,19].

This post hoc analysis includes the data of all 150 patients randomized to the eHealth-adjusted care pathway arm.

### Ethics Approval

All patients provided written informed consent, which was obtained during the first study visit at the HF outpatient clinic before any study procedure was undertaken. The study was approved by the Medical Ethics Committee of the University Medical Center Utrecht (number 12/456), and the e-Vita HF trial was registered on ClinicalTrials.gov (NCT01755988).

### Patient Population

Patients were eligible if they were 18 years of age or older, had an established diagnosis of HF for more than 3 months, were managed at a participating HF outpatient clinic, and had sufficient cognitive function [1,19]. Exclusion criteria were as follows: (1) nonavailability of internet and email; (2) inability of the patients or their family to work with internet and email; and (3) inability of the patients, their family, or caretakers to read and understand Dutch [1,19].

### Components of the eHealth-Adjusted Care Pathway

In the eHealth-adjusted care pathway, an interactive platform for HF disease management (the e-Vita platform) was used. The e-Vita HF platform consisted of a remote patient monitoring platform for biometric values plus a medical and health information website (ie, personal health record), and therefore, it was not integrated in a mobile phone app.

When logging on for the first time to the personal health record, every patient saw a pop‐up with a brief explanation about the website and the services that could be found on there. After the pop‐up, every patient was directed to the home page. From there, patients were able to access all

functionalities of the website. It consisted of the following set of interrelated services, which could be accessed via the home page: (1) self‐monitoring personal health values, where patients could view (previous) biometric values and, if needed, manually add extra measurements in addition to the values received by Bluetooth (eg, blood pressure, pulse, and weight); (2) the website heartfailurematters.org was also a feature of the home page (with a smooth operating link to the freely accessible website); (3) medicine chart, where patients could add their medication; and (4) account settings, where patients could change personal information.

In addition, the specialist HF nurses instructed the patients and their caregivers on how to use the remote patient monitoring elements of the e‐Vita platform, including guidance on the heartfailurematters.org website. Patients learned to record biometric measurements (ie, weight, blood pressure, and pulse) on a fixed time point every day. Blood pressure and pulse were measured with the same device (a Bluetooth-enabled electronic blood pressure monitor). Weight was measured on a Bluetooth-enabled scale. The measurements were automatically forwarded to the e‐Vita website with Bluetooth. If recordings of weight, blood pressure, or pulse were outside of personally adjusted limits (to reduce redundant alerts) or if measurements were not recorded, the specialist HF nurse received an alert via the e-Vita platform. If deemed necessary, the nurse contacted the patient by phone to explore symptoms and, if needed, adjusted the individual management or asked the patient to visit the outpatient clinic. The study team and help desk of the e-Vita platform were available by phone and email during office hours to provide help when patients or health care professionals experienced technical problems with the e-Vita HF platform or any other technical issue.

### Measurements

#### Demographic and Disease-Specific Characteristics

Demographic and disease-specific characteristics were collected from electronic patient records at baseline. The NYHA class was patient reported. Self-care behavior was measured at baseline and after 12 months with the European Heart Failure Self-care Behaviour Scale. It consists of 9 items that were scored on a 5-point Likert scale with standardized scores from 0 to 100 and with a higher score meaning better self-care [20,21].

#### Participants’ Use of Log Data of the Personal Health Record

When logging onto the web-based personal health record, every patient had to tick off a box to accept the general conditions, including a paragraph about tracking their use of the personal health record for research purposes. With accepting these general conditions, patients gave permission for collecting their usage data. The developers of the personal health record facilitated the collection of log data. All data were stored and processed following the actual privacy regulations. The log data of the personal health record were collected from October 9, 2013, to December 25, 2015.

For every patient, sessions (ie, the actions taken between logging on and logging out) were identified first. All actions that were performed within half an hour after the last action were considered to be part of the same session [22].

During the e-Vita HF study, the following log data were collected: (1) the time and date of the action, (2) identification of the action, and (3) optional additional information (eg, what information was viewed by the patients, or which personal goal was added).

The log data were divided into 2 time periods, that is, 0-6 and 6-12 months from baseline, to describe the change over time of the use of features of the personal health record other than the home page. The log data used were as follows: (1) clicking on “enter biometric measurements,” (2) views of “graphs biometric measurements,” (3) opening “previous measurements,” (4) opening “my target biometric values,” (5) opening “disease information,” (6) opening “my medication,” and (7) clicking on “add medication.”

Because log data do not provide information concerning who used the personal health record (patient or caregiver), we analyzed the question “How many times have your family, friends and/or caregivers visited the personal health record on average in the past 3 months?” from the self-administrated “use of personal health record” questionnaire (measured at 3, 6, and 12 months). The questionnaire consisted of 17 questions, which were scored on an 8-point Likert scale (1=never and 8=daily).

#### Log Data of Remote Patient Monitoring

Log data were collected on weight, blood pressure, and pulse. The developers of the e-Vita heart failure remote patient monitoring system facilitated the collection of log data. All data were pseudonymized, stored, and processed following the current privacy regulations.

The log data of the biometric measurements were collected from October 9, 2013, to December 25, 2015. During the e-Vita HF study, the following log data of remote patient monitoring were collected: the date, time, values, alert triggers, and problems with measurements.

The population was divided into adherent and nonadherent based on adherence to weight transmission to assess whether patient characteristics were related to adherence or nonadherence to weight transmissions. Because there is no “gold standard” measure for telemonitoring adherence, we defined adherence as transmitting weight ≥3 times per week for at least 42 weeks in 1 year (ie, 80% of the time). Most patients mentioned to be in NYHA class I at the start of the study. In addition to the fact that all participants were in a stable phase of their disease, we did not define adherence as daily transmitting weight but as at least 3 times a week transmission of weight, similar to what the “Telemonitoring to Improve Heart Failure Outcomes” study used (intended use of 3 times per week) [14]. We used the criteria of at least 42 weeks, since patients did not use remote patient monitoring during hospitalizations and holidays.

We measured adherence for the complete set of biometric measurements (blood pressure, pulse, and weight) and for each biometric measurement separately. We also compared the adherent and nonadherent patients with regard to hospitalizations the year before participation and during the study as well as their self-care behavior based on the European Heart Failure Self-care Behaviour Scale at baseline and at the end of the study (ie, 12 months).

### Statistical Analyses

Descriptive statistics were used to describe the actual use of the e-Vita HF home page by patients during 1 year. To univariably compare the demographic and clinical characteristics between patients adherent and nonadherent to weight transmissions, the chi-square test was used for categorical variables, the independent 2-tailed *t* test was used for continuous variables in case of normal distributions, and the Mann-Whitney *U* test was used in case of skewed distributions. Nominal variables were expressed as n (%). Continuous variables were expressed as means with SDs or medians with IQRs. Data were extracted to IBM SPSS Statistics for Windows (version 26; IBM Corp) for statistical analysis.

## Results

### Demographic and Disease-Specific Characteristics

The mean age of the 150 patients studied was 67 (SD 11) years, and 25% (n=37) were female. The mean left ventricular ejection fraction was 36% (SD 11%), and the majority were in self-reported NYHA class I and II (I: n=49, 46%; II: n=54, 36%; III: n=18, 12%; and IV: n=9, 6%).

### Actual Use of Remote Patient Monitoring Over Time (Transmission of Blood Pressure, Pulse, and Weight)

In a period of a year, 111 (74%) patients were adherent to weight transmissions (Table 1), and 101 (67%) were adherent to transmitting data on all three biometric values. Individual adherence showed to be dynamic, changing over time.

**Figure 1:**
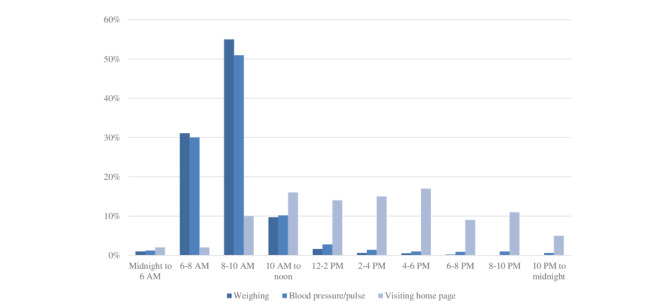
Percentage of patients adherent to remote monitoring per month.

In the first 6 months, patients were most adherent to weight transmissions (n=129, 86% vs n=109, 73% between 6 and 12 months; Figure 1). The percentage of patients adherent to remote patient monitoring per month varied over time (Figure 1). Weight transmission varied between 95% (n=143) and 81% (n=122), blood pressure and pulse between 91% (n=137) and 73% (n=110), and all three (ie, weight, blood pressure, and pulse) between 91% (n=137) and 71% (n=107) per month over the period of 1 year.

A total of 6 (4%) patients never used the e-Vita HF platform (ie, remote patient monitoring and e-Vita HF website). Another 2 (1%) patients never used the remote patient monitoring facilities; they only logged on to the personal health record during follow-up.

**Figure 2:**
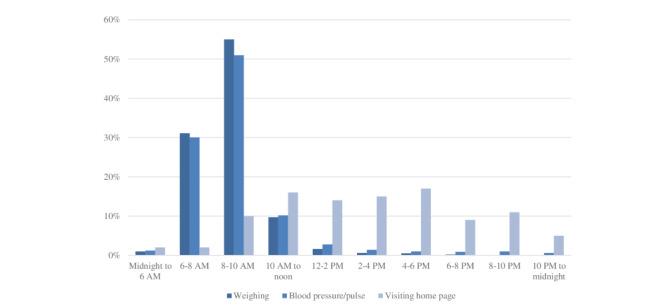
Time of the day for weighing, blood pressure and pulse measurements, and visiting the home page of the electronic personal health record.

In total, 85% (128/150) of the measurements were done between 6 and 10 AM, and during that period, 26% (39/150) of patients visited the home page of the personal health record (Figure 2). Transmitting biometric values did not differ between the weekdays or the different months of the year.

**Table 1:** Baseline characteristics of the 150 patients divided into adherent and nonadherent to weighing during a year. The italicized *P* value is significant.

Characteristics			Adherent^a^ (n=111)		Nonadherent (n=39)		*P* value	
**Demographics**								
	Age (years), mean (SD)	67.3 (9.9)		64.8 (13.6)		.23		
	Female, n (%)	29 (26)		8 (20)		.20		
**Educational level, n (%)**								.39
	Low	24 (22)		10 (26)				
	Middle	54 (45)		14 (45)				
	High	33 (30)		15 (39)				
Living alone, n (%)			25 (22)		10 (26)		.21	
BMI, mean (SD)			27.8 (5.0)		28.2 (7.0)		.67	
Current smoking, n (%)			12 (11)		9 (23)		.06	
**Comorbidity**								
	ACS^b^, n (%)	51 (46)		21 (54)		.40		
	Atrial fibrillation, n (%)	50 (45)		16 (41)		.66		
	Hypertension, n (%)	45 (40)		20 (51)		.24		
	Diabetes mellitus, n (%)	31 (28)		9 (23)		.56		
	COPD^c^, n (%)	26 (23)		10 (26)		.78		
	Depression, n (%)	23 (21)		3 (8)		.06		
	Anxiety disorder, n (%)	18 (16)		5 (13)		.61		
**Heart failure and clinical characteristics**								
	Duration of HF^d^ (months), median (IQR)	25 (12-51)		33 (11-59)		.48		
	LVEF^e^, mean (SD)	36.4 (11.2)		34.9 (10.9)		.47		
	Hospitalization due to HF in the 6 months before the start of the study, n (%)	9 (8)		9 (23)		*.02*		
**NYHA^f^ class at baseline, n (%)**								.87
	I	51 (46)		18 (46)				
	II	40 (36)		14 (36)				
	III	11 (10)		7 (18)				
	IV	9 (8)		0 (0)				
**Questionnaires**								
	EHFScBS^g^ total score, median (IQR)	72 (61-83)		72 (64-80)		.97		

^a^Adherent with weight transmissions ≥3 times a week for at least 42 weeks in 1 year.

^b^ACS: acute coronary syndrome.

^c^COPD: chronic obstructive pulmonary disease.

^d^HF: heart failure.

^e^LVEF: left ventricular ejection fraction.

^f^NYHA: New York Heart Association.

^g^EHFScBS: European Heart Failure Self-care Behaviour Scale.

### Clinical Characteristics of Patients Adherent to Weight Transmissions

The mean age of the 111 adherent patients was 67 (SD 10) years, and 29 (26%) were female (Table 1). Patients adherent to weighing were less often hospitalized for worsening of HF in the 6 months *before* enrollment in the study (adherent patients: n=9, 8% vs nonadherent patients: n=9, 23%). There was no difference between the adherent and nonadherent patients in the number of hospitalizations during the study; in the adherent group, 4 (3.6%) patients had more than one hospitalization compared to 1 (2.6%) patient in the nonadherent group. Furthermore, there was no difference in the total European HF Self-care Behaviour Scale scores between the groups at baseline or at 12 months.

### Use of the Personal Health Record

**Figure 3:**
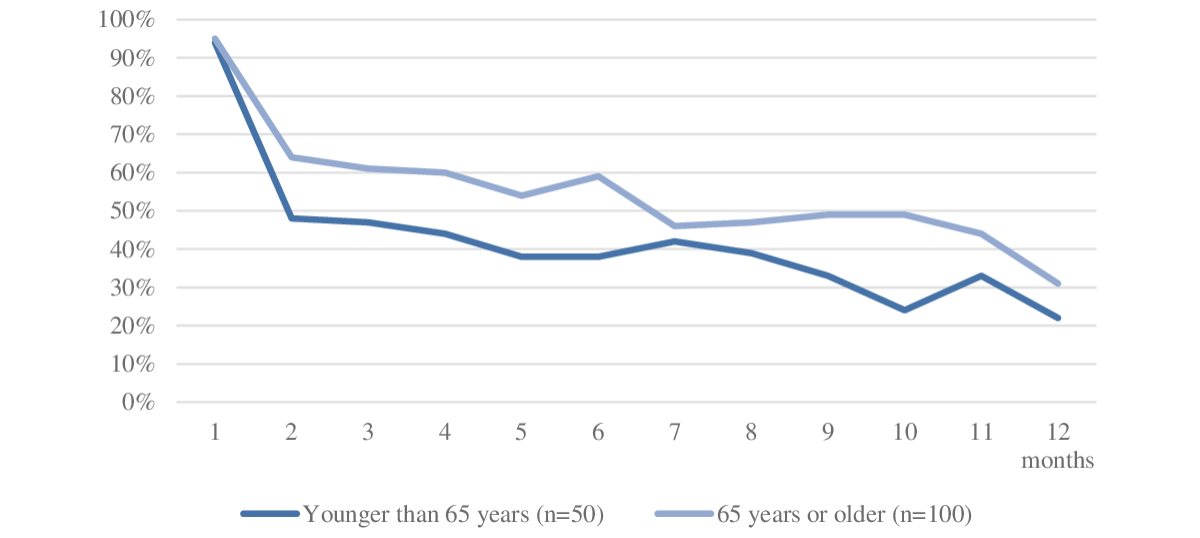
Decline of over-the-year visits to the home page of the electronic personal health record of patients <65 years and patients ≥65 years of age.

In the first month, 142 (95%) patients visited the home page of the personal health record with a median number of 21 (IQR 7-54) visits. The number of patients visiting the home page declined over time, most rapidly in the first 2 months of follow-up (Figure 3).

A total of 20 (13%) caregivers visited the personal health record at least once during 12 months. There was no difference between caregivers of adherent patients and caregivers of nonadherent patients.

The home page was most frequently visited in December (n=20, 13% of the visits) and least frequently visited in June (n=6, 4%); it was visited most often on Wednesdays (n=26, 17%) and less often on weekends (both weekend days; n=15, 10% of the visits). This was not related to the month or day of enrollment in the study. Between 10 AM and 6 PM, the home page was most often visited (n=93, 62% of the visits; Figure 2). Over time, older patients logged on to the personal health record more often than younger patients (Figure 3). There was no difference in sex related to logging on to the home page of the platform.

**Figure 4:**
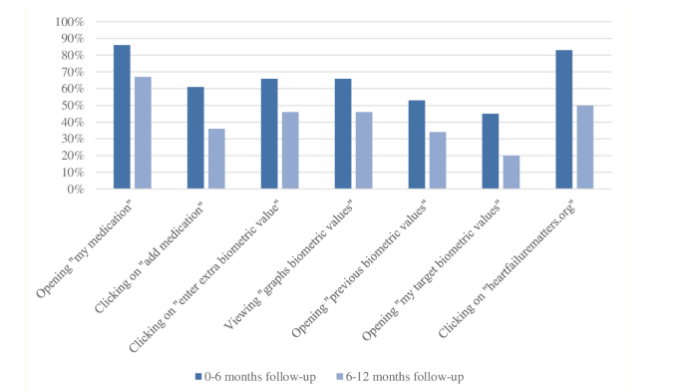
Percentage of patients visiting features of the electronic personal health record 0-6 months versus 6-12 months of follow-up (n=150).

In the first 6 months, the most frequent action on the personal health record—after visiting the home page—was “opening my medication” (n=129, 86% of the patients), followed by visiting the heartfailuremattters.org website (n=125, 83% of the patients; Figure 4). In the last 6-12 months of follow-up, the personal health record was less used compared to the first 6 months.

Patients adherent to weight transmissions visited the home page of the personal health record significantly more often in the last 3 months of follow-up compared to nonadherent patients (n=71, 64% vs n=8, 20%; *P*<.001).

## Discussion

### Principal Findings

We quantified the use of telemonitoring in patients with stable HF and assessed whether patient characteristics correlated with adherence or nonadherence to weight transmissions. Overall, adherence to transmitting biometric values (ie, weight, blood pressure, and pulse) was high, ranging from 90% (n=135) at the start of the study to 71% (n=107) after 12 months. In addition, the use of personal health record was high at the start (n=143, 95%) but dropped to 25% (n=38) at 12 months.

Adherence to weight transmissions was rather high (on average n=111, 74% over the year), with the highest adherence during the first 6 months (n=131, 87%).

Of note, patients adherent to weight transmissions were less often hospitalized for HF in the 6 months before enrollment in the study and nonsignificantly so during the follow-up period of the study. This finding may be a coincidental finding but may also be related to the “healthy adherer effect.” In any case, the main reason for using telemonitoring in patients with stable HF may not be the reduction of HF hospitalizations but to safely reduce routine health care utilization, including face-to-face contacts with a specialist HF nurse.

Clinical and demographic variables seemed not related to adherence to transmitting weight. This is in line with a recent systemic review that concluded that symptom severity, comorbidity, sex category, and marital status were inconclusively associated with better adherence to telemonitoring for any of these factors [23].

Monitoring vital signs is a key component of self-care for patients with HF, but the relationship between self-care behavior and age, gender, education, and left ventricular ejection fraction values is inconsistent [24].

The decline of adherence over time is a common finding with transmitting biometric values, and as such, it is also reported in previous telemonitoring HF studies [25,26]. Adherence rates in remote patient monitoring ranged from 40% to 90% in previous eHealth heart failure studies, which is similar to our study [6,14,26-32]. Interpreting our results, it is important to realize that adherence to remote patient monitoring is dependent on the telemonitoring system used, such as interactive voice response–based interventions and eHealth apps on mobile phones that leverage devices already familiar to patients [27]. Second, the remote patient monitoring interventions vary in intensity of contacts with HF professionals, for example, remote patient monitoring on top of usual care and remote patient monitoring including interactive and intensive coaching modules. Third, adherence is defined and measured inconsistently across studies and in diverse patient populations. In our study, we used a definition similar to the one the “Telemonitoring to Improve Heart Failure Outcomes” study used (intended use of 3 times per week) [14]. To correct the number of weeks for holidays and hospitalizations, we used an adherence percentage of 80% over 1 year (ie, 42 weeks). Fourth, adherence is a dynamic measure that often changes within patients. Adherence can vary per day, week, or month, and it is influenced by social and economic factors as well as factors that are related to the health care system, the condition of the patient, therapy, and other factors related to the patient [33].

Adherence to weight transmission was higher than adherence to transmitting blood pressure and pulse. This is in line with the “Trans-European Network–Home-Care Management System” study [31]. One of the reasons can be that weighing was already a habit for patients before the start of the study, whereas measuring blood pressure and pulse were not. Furthermore, measuring blood pressure is more time consuming than weighing and can be unpleasant; it may give a tinkling feeling in the arm and hand. Another explanation might be that patients had insufficient knowledge on the relevance of these parameters. Moreover, patients who are stable might not see any changes over time or a direct link between small changes in blood pressure and heart rate and their symptoms. Finally, an explanation could be that patients with HF in NYHA class I-II may feel less urgency to monitor the worsening of HF because they remain stable over a long period of time and may not experience substantial limitations due to their HF in their daily life. An important finding in this study regarding the use of a personal health record is that patients did not look at their (previous) biometric measurements very often, and therefore, it seems that most of the patients did not really use the personal health record for monitoring their own HF. This can be partly explained by the personal health record being mainly introduced in light of the evaluation study, and the training of the HF nurses predominantly focused on how to collect the data for this study. As a result, HF nurses did not know what was expected from them with regard to using the services of the personal health record. This caused HF nurses to find it difficult to motivate their patients in using the personal health record. Importantly, however, limited use of the personal health record is a rather common finding in eHealth studies [34-36]. Several systematic reviews focusing on the implementation of complex telemonitoring interventions and personal health records stress that the (perceived) fitting of telemonitoring technologies within the current working routines and the interoperability with other systems are key factors for a successful implementation [36-39].

In this study, a digital platform with automatic transmission was used, whereas a mobile phone app may be easier to use by patients.

### Limitations

The study sample was rather small and too small for multivariable regression analysis, and this post hoc analysis of the e-Vita HF trial was observational in nature. In addition, we were unable to account for days when patients experienced technical problems with the remote patient monitoring equipment. However, technical problems occurred rarely and could be solved by a help desk we had in place. Therefore, it is unlikely that technical problems affected the degree of adherence. Nevertheless, this is one of the few studies that both evaluated remote patient monitoring and personal health record in patients with HF.

### Conclusions

Adherence to transmitting biometric values was high among stable outpatients with HF who were participating in an eHealth study, and it was best for weight; however, adherence decreased over time. Clinical and demographic variables seem not related to adherence to transmitting weight.

## Abbreviations

HF: heart failure
NYHA: New York Heart Association

## Data Availability

The data used to support the findings of this study are available from the corresponding author upon reasonable request.
